# Fast Mode Decision for 3D-HEVC Depth Intracoding

**DOI:** 10.1155/2014/620142

**Published:** 2014-05-19

**Authors:** Qiuwen Zhang, Nana Li, Qinggang Wu

**Affiliations:** College of Computer and Communication Engineering, Zhengzhou University of Light Industry, Zhengzhou 450002, China

## Abstract

The emerging international standard of high efficiency video coding based 3D video coding (3D-HEVC) is a successor to multiview video coding (MVC). In 3D-HEVC depth intracoding, depth modeling mode (DMM) and high efficiency video coding (HEVC) intraprediction mode are both employed to select the best coding mode for each coding unit (CU). This technique achieves the highest possible coding efficiency, but it results in extremely large encoding time which obstructs the 3D-HEVC from practical application. In this paper, a fast mode decision algorithm based on the correlation between texture video and depth map is proposed to reduce 3D-HEVC depth intracoding computational complexity. Since the texture video and its associated depth map represent the same scene, there is a high correlation among the prediction mode from texture video and depth map. Therefore, we can skip some specific depth intraprediction modes rarely used in related texture CU. Experimental results show that the proposed algorithm can significantly reduce computational complexity of 3D-HEVC depth intracoding while maintaining coding efficiency.

## 1. Introduction

Three-dimensional video (3DV) depth-enhanced format has gained increasing interest recently. A depth map represents 3D scene information and is commonly used for depth image-based rendering (DIBR) [1] to support 3D television (3DTV) [2] and free viewpoint television (FTV) [3] applications. The virtual view is generally rendered by the DIBR technique and its quality depends highly on the quality of depth map. Thus, efficient depth map compression has been one of the significantly important issues to realize the 3D video coding. Since the characteristic of a depth map is very different from that of a texture video, use of existing codec to compress depth maps will introduce distortions into the novel virtual views. To this end, efficient depth map coding is currently being investigated by the Joint Collaborative Team on 3D video coding extension development (JCT-3V), with the goal of developing new standards of high efficiency video coding based 3D video coding (3D-HEVC) [4].

3D-HEVC exploits depth modeling mode (DMM) [5] for a better coding of object edges in depth maps; four new intraprediction modes for depth coding are added. And traditional high efficiency video coding (HEVC) uses 35 different intraprediction modes for each prediction unit. Consequently, the depth intraprediction part in 3D-HEVC, which consists of DMM and HEVC intraprediction mode, becomes the most computationally intensive part in a 3D video depth coding system. Huge amount of depth maps data and ultrahigh computational complexity make 3D-HEVC encoder difficult to be realized in real time. Therefore, it is necessary to develop a method that can reduce depth intraprediction complexity of 3D-HEVC with minimal loss of image quality.

Recently, studies on reduction of computation complexity of depth map coding have been reported. A depth block skip algorithm is proposed in [6] to reduce computational complexity by forcing the coding of a depth map block in SKIP mode. A joint of texture video and depth map motion estimation is proposed in [7] to reduce the complexity of depth map coding. An early mode termination strategy is proposed in [8] based on the difference between the current macroblock (MB) and the colocated MB in the original frame. A fast depth map coding has been made in our previous work [9] based on sharing motion vector and SKIP mode from the texture video, which can highly reduce the depth map coding complexity. The aforementioned methods are well developed for depth map coding achieving significant time savings. However, these methods are not directly applicable to the 3D-HEVC. Since there are more reference frames, the prediction process of 3D-HEVC is more complex than before. Fast mode decision for depth intraprediction in 3D-HEVC is a new topic.

Some fast algorithms for reducing the 3D-HEVC depth intraprediction computational complexity are proposed in [10, 11]. In [10], a fast mode decision algorithm to early terminate the DMM full rate-distortion (RD) cost calculation is proposed in 3D-HEVC. In [11], a complexity reduction method for depth modeling mode 3 (DMM 3) based on the corresponding texture block is proposed to reduce the number of wedgelet candidates. However, in those methods, motion information and prediction mode correlations between the texture video and the depth map are not efficiently utilized. This situation results in a limited time saving. To overcome these problems, this paper proposes a fast intramode decision algorithm for 3D-HEVC depth map coding, which takes advantage of the texture characteristic and prediction mode correlation between the texture video and the corresponding depth map. The proposed fast intramode decision algorithm consists of two approaches: the early termination mode decision and the adaptive depth map coding unit (CU) depth range determined based on related texture video. Experimental results show that the proposed algorithm can save about 46% computational complexity on average while maintaining coding efficiency.

The rest of this paper is organized as follows. In Section 2, we analyze the correlation of the texture characteristic and prediction mode correlation between the texture video and the corresponding depth map and propose two efficient intramode decision approaches.  Section 3 compares performances of the individual approaches as well as a combination of all the two approaches.  Section 4 concludes this paper.

## 2. Proposed Fast Intradecision for Depth Map Coding

During the 3D-HEVC intraprediction process (similar to HEVC), the intramode decision process in 3D-HEVC is performed using all the possible intraprediction modes to find the one with the least rate distortion (RD) cost using Lagrange multiplier. However, this technique will result in extremely large computational complexity and limit the use of 3D-HEVC encoders in practical applications. Therefore, fast algorithms, which can reduce the complexity of intradecision without compromising coding efficiency, are very desirable for real-time implementation of 3D-HEVC encoders.

### 2.1. Early Termination Mode Decision

In the test model of 3D-HEVC, the depth intraprediction modes include DMM and HEVC intraprediction mode. DMM is adapted to the specific characteristics of depth map by applying nonrectangular block partitions for approximating the signal of a depth block containing an edge. The depth map is mainly characterized by sharp object edges and large areas of nearly constant or slowly varying smooth regions. Thus, most of the CUs in depth maps are smooth regions, and DMM is designed for depth map CU with sharp edges transition which is less efficient for smooth depth map CU coding. We test depth maps of eight 3D video sequences,* Kendo*,* Balloon*,* Newspaper*,* Shark*,* Undo*_*Dancer*,* GT_Fly*,* Poznan*_*Street,* and* Poznan*_*Hall2,* with a 3D-HEVC encoder as statistical analysis of the DMM selection rate as best mode in depth map intracoding. Statistical results of depth maps are shown in Table 1.

The result shows that most of CUs in intraslices of depth maps choose HEVC intraprediction mode as the optimal mode: the average percentage of choosing HEVC intraprediction mode is about 99%. It also can be seen in Table 1 that the percentage for a CU to be coded in DMM is about 1%, and DMM has very low probability to be selected as best mode in 3D-HEVC depth intracoding. On the contrary, the costs of computing the RD costs of DMM modes are much higher than those of intramodes. The small probability of DMM suggests that the practice of deciding the best intramode for all the possible intraprediction modes may have a certain limit in reducing 3D-HEVC computational complexity. If we can decide in advance whether the intramode for a depth CU is DMM or not, the wasting process of computing the RD cost for inefficient modes can be omitted, and thus a huge amount of computation for intramode size decision can be saved in 3D-HEVC depth map coding. Therefore, it is necessary to propose an early termination (ET) mode decision in 3D-HEVC depth intracoding approach. In the following, we will detail the two ET algorithms.

*Strategy 1: ET Based on the Homogeneity Checking.* In 3D-HEVC encoders, DMM is utilized together with HEVC intraprediction for depth map coding. During the intramode decision process, 3D-HEVC uses the combination of the rough mode decision (RMD) and RD optimization (RDO) to select the best intradirection, a full RD search list is created, and* N* best modes in RMD (3 for 64 × 64, 32 × 32, and 16 × 16 prediction unit (PU) sizes, 8 for 8 × 8 and 4 × 4 PU sizes) are selected from 36 intraprediction modes for full RD cost calculation. After the selection of the first* N* best candidate modes based on the RMD process, all the DMM are also added to the full RD search list. This achieves the highest coding efficiency but requires a very high computational complexity. Since depth maps have large areas of nearly constant and slowly varying homogeneity regions, it is not efficient to use a full RD search for a whole depth map. In fact, small PU sizes are suitable for treeblocks in the homogeneous region, and large PU sizes are chosen for treeblocks with rich textures region [12]. We can see from experiments of depth intracoding that the sharp object edge occurs very frequently for large rich region coding. On the other hand, the sharp object edge is rarely chosen for treeblocks with homogeneous textures region. These results show that 3D-HEVC depth intramode search should be adaptively determined based on the homogeneity checking of treeblocks.

Table 2 shows the correlation of PU size distributions among DMM. It is observed that treeblocks in DMM of depth maps choose size 8 × 8 and 4 × 4 as the optimal PU size: the average percentage of choosing size 8 × 8 and 4 × 4 is about 90%. It also can be seen in Table 2 that the percentage for a treeblock to be coded in other PU sizes such as 64 × 64, 32 × 32, and 16 × 16 is very low, about 10%. If we can decide in advance whether the PU size for a depth treeblock is 8 × 8 and 4 × 4 or not, the wasting process of computing the RD cost for inefficient modes can be omitted, and thus a huge amount of DMM computation for mode size decision can be saved. Thus, it is better to have a proper ET strategy from the midway of the fast mode decision algorithm for 3D-HEVC depth intracoding. The proposed ET algorithm is made as follows: for depth treeblocks in 3D-HEVC intramode decision, only PU size 8 × 8 and 4 × 4 is tested for DMM, and the other PU size only selects HEVC intraprediction mode.

*Strategy 2: ET Based on the Texture Video-Depth Map Correlation.* In general, through the mode maps of encoded frames, one can approximately learn the motion and texture characteristics of those video frames [13]. On the other side, two frames of the same time instant from the texture video and depth map correspond to the same content with similar video characteristic, resulting in a large correlation of depth treeblocks (such as motion vectors and object edges). The texture characteristic of a treeblock in the depth map is similar to that of the corresponding treeblock in the texture video. On the basis of these observations, we propose using the texture characteristics of the encoded texture video to analyze the 3D-HEVC intraprediction mode distribution of depth maps. In order to utilize the correlation between the texture video and the corresponding depth map, we define a set of predictors (*Ω*) for a treeblock in depth maps as follows:

(1) $$\begin{array}{c} \Omega =\left\{C_{1},C_{2}\right\}, \end{array}$$

where *C*_1_ denotes the difference between maximum and minimum pixel values of the related texture video treeblock and *C*_2_ denotes the different constant pixel regions of the related texture video treeblock.

A threshold based on related texture video image is used to achieve ET for different depth intramodes, which makes it content dependent. The threshold (*Tr*⁡) is set to the average of the texture characteristic of related texture video as shown in

(2) $$\begin{array}{c} \mathrm{Tr}=\alpha \cdot \omega_{1}+\beta \cdot \omega_{2}, \end{array}$$

where *α* and *β* are the weight factors and *ω*_1_ and *ω*_2_ are defined according to the effect of related texture treeblock on current depth treeblock. When the difference pixel values (between maximum pixel and minimum pixel in a related texture treeblock) is less than 10 (*C*_1_ ≥ 10), *ω*_1_ ≥ 1; and when at least two different constant pixel regions in a related texture treeblock (*C*_2_ ≥ 2), *ω*_2_ ≥ 1. Based on extensive experiments, the weight factors *α* and *β* are set to 0.3 and 0.7, and the threshold *Tr*⁡ is set to 1, which achieves a good and consistent performance on a variety of test sequences with different texture characteristics. The criterion is given as follows: when the minimal cost of a candidate is smaller than *Tr*⁡, terminate the procedure of intramode decision at current depth treeblock; the depth intraprediction mode only selects HEVC intraprediction mode but not DMM.

To verify legitimacy of the proposed two ET methods, extensive simulations have been conducted on a set of video sequences as listed in Table 3. By exploiting the early termination mode decision in 3D-HEVC under the aforementioned test conditions in Table 1, we investigate the effectiveness of the proposed ET methods. Table 3 shows the accuracies of the proposed ET methods. The average accuracy of the proposed ET based on the homogeneity checking is larger than 81%. The average accuracy of the proposed ET based on the texture video-depth map correlation method achieves 90% with a maximum of 95.8% in “Newspaper” and a minimum of 84.6% in “Poznan_Street”. The accuracies of the proposed ET methods are consistent for all test sequences with different properties. The results shown in Table 5 indicate that the proposed ET methods can accurately reduce unnecessary intraprediction modes in 3D-HEVC encoder.

### 2.2. Adaptive Depth Map CU Depth Range (DR) Determined Based on Related Texture Video

3D-HEVC utilizes the quadtree-structured coding unit structure introduced in HEVC [14] for both the texture video and depth map compression. In the current test model of 3D-HEVC, CU depth has a fixed range for a whole texture video and depth map sequence. Similar to the joint model of HEVC, a complex RDO process in 3D-HEVC intracoding is performed using all the possible depth levels and prediction modes of the quadtree to find the one with the least RD cost using Lagrange multiplier, and the 3D-HEVC makes this “try all and select the best” method for both texture video and depth map and encodes both treeblocks separately. However, this technique will result in high computational complexity and limit the use of 3D-HEVC encoders in real-time applications. Since the texture video and its associated depth map represent the same scene, it is a high correlation among the prediction mode from texture video and depth map. The exhaustive depth map intracompression is inefficient, and depth map CU depth range should be adaptively determined based on the related texture video treeblock. A depth quadtree prediction is proposed in [15] based on the collocated quadtree in the texture video. However, this method simply utilizes the partition information from the texture video treeblocks, and most of depth map treeblocks still need to perform on more than three depth levels. Therefore, the time saving of the method in [15] is rather limited.

**Figure 1:**
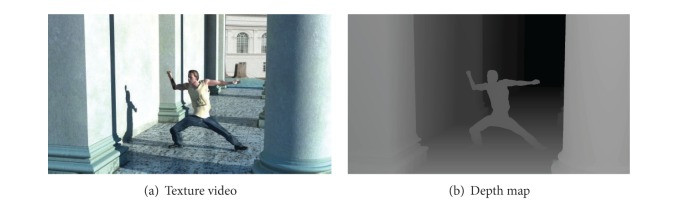
Texture video and corresponding depth map.

The depth map represents the distance from cameras to objects, which has characteristics of both the texture video signal and data on *z*-axis in world coordinate. Therefore, the texture video and its related depth map represent the same scene. In general, the texture video and the depth map have similar characteristics. As shown in Figure 1, boundaries of the depth map have similar shape with that of the texture video, and directions of object movements are the same in both texture and depth video coding. It is a high probability that quadtree CU structure from the texture video and the depth map is like each other. Meanwhile, the depth level of colocated texture video treeblock affects the depth level determination process of the depth map treeblock; the optimal depth level of a depth map treeblock is the same as or very close to the depth level of its texture video treeblock. Thus, we can make use of the texture video and depth map correlations to analyze region properties and skip certain depth level on unnecessary depth map CU sizes.

In the test model of 3D-HEVC, the CU (texture video and the depth map) depth level has a fixed range for a whole video sequence. Since the optimal depth map CU depth level is highly correlated with its colocated texture video, it is not efficient to use all depth levels. We can determine depth map CU depth range and skip some specific depth levels rarely used in 3D-HEVC depth map intracoding. In fact, small depth values tend to be chosen for CUs in the homogeneous region, and large depth values are chosen for CUs with complex region [16]. Based on this concept, we can force the encoder to limit the partitioning of the depth map at the same depth level as the partitioning of the texture video. However, in this method, the prediction mode correlations between the color video and the depth map are not efficiently utilized (the depth map simply utilizes the partition information from the texture video), and most of depth map treeblocks still need to perform on more than three depth levels. This situation results in a limited time saving. Since texture video has more edges due to illumination changes, patterned textures, and shadows, it is thus in general more partitioned than depth map in complex region. Thus, the texture video space is classified into two regions based on the texture characteristic: homogeneous region and complex region; the treeblocks classification can be represented by the following equations:

(3) RC≤T treeblock∈homogeneous  regionRC>T treeblock∈complex  region,

where RC means the image regions complexity and *T* depends on local characteristics of the video image.

To verify legitimacy of this method (which simply utilizes the partition information from the texture video), extensive simulations have been conducted on a set of video sequences as listed in Table 4. Table 4 gives the prediction mode accuracy of this method for the treeblocks with homogeneous region and complex region. It can be seen from Table 4 that, for the treeblocks with homogeneous region, the average accuracy of this method is from 90% to 99%. The results indicate that this method can accurately reduce unnecessary depth level in homogeneous region. For the treeblocks with complex region, the average accuracy of this method is from 71% to 89%. It can be seen from statistical results in Table 4 that this method holds particularly well for the “Shark”, “Undo_Dancer” and “GT_Fly” sequences because they are computer-generated sequences with ground-truth depth maps. Correspondingly, the other depth maps considered are estimated using depth estimation algorithms; they may contain errors. The depth maps associated with texture images are usually not the ground truth because existing depth estimation methods still have difficulties in generating accurate depths at object edges or in areas with less texture. Distortion may occur during depth map estimation, which will result in noisy depth map. These noisy sometimes come out as false edges in the depth map which cause a fine partitioning of an area that is supposed to be flat. These false edges are smoothed out at lower bitrates, which is why the accuracy percentage decreases from QP34 to QP45. Thus, we can conclude that a treeblock with homogeneous region is much more accurate compared with the treeblock with complex region. During our experiments on various video sequences using 3D-HEVC encoders, we observe that most depth map CUs in the complex region have been decided as the small depth values rather than its colocated texture video CU depth level, while only depth map CUs in the homogeneous region need the same depth values in texture video CU depth level. Thus we can predetermine a depth map CU depth level in the complex region; if the texture is split at depth value, the depth map CU is only allowed to be split at depth level. As a result, the candidate intraprediction modes are limited to a small subset, and the computational complexity could be highly reduced.

Table 5 shows the accuracies of the proposed algorithm in complex region. The average accuracy of the proposed algorithm is larger than 84% with a maximum of 96% in “QP = 45” and a minimum of 84% in “QP = 34”. The results shown in Table 5 indicate that the proposed algorithm can accurately reduce unnecessary depth map CU depth level in complex region. Based on this statistical tendency, this paper proposes an adaptive depth map CU depth range algorithm that utilizes the coded texture video quadtree to control the depth map CU depth range and coding construction. The proposed adaptive depth map CU depth range algorithm is made as follows: for the treeblocks with homogeneous region, the depth map CU depth level equals its colocated texture video CU depth level; for the treeblocks with complex region, the depth map CU depth level is less than its colocated texture video CU depth level.

## 3. Experimental Results

We evaluate the proposed fast mode decision algorithm for 3D-HEVC depth intracoding, which is implemented on a 3D-HEVC Test Model (HTM ver. 5.1) encoder. We give the results that followed the intra-only configuration released by JCT-3V [17]. Detailed encoding parameters for the reference software are shown in Table 6. After encoding, the intermediate rendered views were synthesized between each viewpoint. The intermediate rendered views are generated at the receiver using view synthesis reference software (VSRS) algorithm provided by MPEG [18]. Rendered PSNR can be measured by comparing the coded rendered view with the image rendered with uncompressed texture video and depth map. The performance of the proposed overall algorithm is compared to the depth intraprediction algorithm for 3D-HEVC and a fast DMM selection algorithm for depth intracoding [10]. In addition, we afford individual performance results of the proposed approaches. For each test sequence, experiments consider a two-view case (C2) and a three-view case (C3). The experimental results are presented in Tables 7 and 8, in which coding efficiency is measured with rendered PSNR and total bit rate (texture video and depth map), and computational complexity is measured with the consumed coding time. Since the proposed approaches affect only depth map intracoding, results for texture video coding are identical; thus the texture video results are not included in the table. The Bjontegaard delta PSNR (BDPSNR) [19] represents the average rendered PSNR gain, bitrate (BDBR) represents the improvement of total bitrates for 3D video coding, and “Dtime (%)” represents the entire coding time change in percentage.

Table 7 shows individual evaluation results of the proposed approaches compared with the original 3D-HEVC algorithm, that is, early termination mode decision (ETMD) and adaptive depth CU depth range determined (AD-CUDRD), respectively. The proposed two approaches can greatly reduce the encoding time with similar encoding efficiency for all sequences. For the ETMD method, 35.9% and 34.9% coding time has been reduced over all sequences under C2 and C3 conditions, respectively. It can be also observed that a consistent gain is obtained over all sequences under both conditions. The coding efficiency loss is very negligible with 0.041 dB–0.049 dB PSNR drop or 1.12–1.22% bitrate increase. This result indicates that ETMD can efficiently reduce unnecessary depth intraprediction modes in 3D-HEVC encoder. As far as the AD-CUDRD method is concerned, 30.5% and 31.4% coding time has been reduced in C2 and C3, respectively, with a maximum of 41% in “Undo_Dancer” (C3) and a minimum of 23% in “Poznan_Hall2” (C2). Meanwhile, the average PSNR drop for all the test sequences is 0.02 dB and the average increase of bitrate is 0.73%, which is negligible. The foregoing result analysis indicates that AD-CUDRD can efficiently reduce the coding time while maintaining nearly the same RD performance as the original 3D-HEVC encoder.

In the following, we analyze the experimental result of the proposed overall algorithm, which incorporates ETMD and AD-CUDRD. The comparison results of the overall algorithm and a state-of-the-art fast intramode decision algorithm (fast DMM selection, FDMMS [10]) for the 3D-HEVC depth coding are given in Table 8. The proposed overall algorithm, respectively, reduces 44.6% and 46.1% coding time under C2 and C3 and achieves the better gain in coding speed compared to FDMMS. Also shown is a consistent gain in coding speed for all test sequences with the lowest gain of 41% for “Poznan_Street” (in C2) and the highest gain of 49% for “GT_Fly” (in C3). On the other hand, the coding efficiency loss is negligible, specifically, where the average PSNR loss is 0.053–0.057 dB or the average increase of bitrate is 1.34–1.38%. Compared with FDMMS, the proposed overall algorithm performs better on all the sequences and achieves more than 18% coding time saving. The coding efficiency losses are negligible considering the time saving it achieves. The proposed overall algorithm has only 0.037 dB PSNR loss or 0.86% bitrate increment compared to FDMMS.

**Figure 2:**
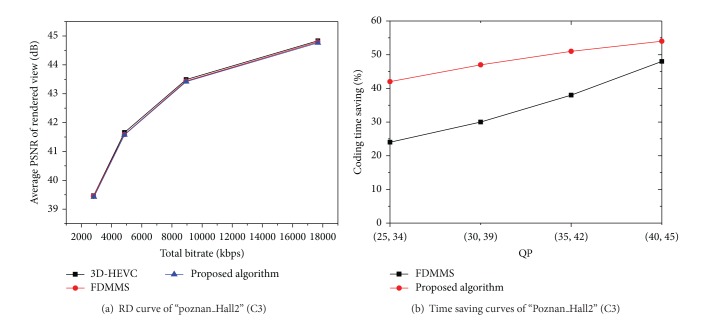
Experimental results of “Poznan_Hall2” (1920 × 1088) under different QPs combinations for texture video and depth map (25, 34), (30, 39), (35, 42), and (40, 45).

Figures 2(a) and 2(b) show the more detailed experiment results under different QPs for “Poznan_Hall2”. As shown in Figure 2, the proposed algorithm can achieve a consistent time saving over a large bitrate range with almost negligible loss in PSNR and increment in bit rate.

## 4. Conclusion

This paper presents a fast mode decision algorithm to reduce the computational complexity of the 3D-HEVC intracoding by exploiting two fast approaches, that is, the early termination mode decision and the adaptive depth map CU depth range determined. The proposed algorithm is implemented on the recent 3D-HEVC reference software. The comparative experimental results show that the proposed algorithm can significantly reduce the computational complexity of 3D-HEVC depth intracoding while maintaining almost the same RD performances as the original encoder and achieves a higher gain time saving compared to the state-of-the-art fast algorithm and FDMMS.

## Figures and Tables

**Table 1:** Statistical analysis of intramode distributions in depth map coding.

Sequences	QP = 34	QP = 39	QP = 42	QP = 45
Kendo	1.0%	0.8%	0.6%	0.5%
Balloons	1.2%	1.1%	0.9%	0.7%
Newspaper	1.1%	0.9%	0.7%	0.9%
Shark	1.9%	1.6%	1.5%	1.3%
Undo_Dancer	0.8%	0.7%	0.5%	0.4%
GT_Fly	2.1%	1.9%	1.6%	1.2%
Poznan_Street	0.7%	0.6%	0.6%	0.5%
Poznan_Hall2	0.2%	0.2%	0.1%	0.1%

Average	1.1%	1.0%	0.8%	0.7%

Experimental condition: temporal length of a GOP = 8, basis quantization parameters (QP) = 34, 39, 42, and 45, treeblock size = 64, and CABAC (context-adaptive binary arithmetic coding) is used for entropy coding.

**Table 2:** Statistical analysis different PU size distributions in DMM.

Sequences	64 × 64	32 × 32	16 × 16	8 × 8	4 × 4
Kendo	None	4.1	6.6	21.1	68.2
Balloons	None	4.4	7.1	15.2	73.3
Newspaper	None	1.6	3.4	12.5	82.5
Shark	None	4.5	8.8	27.3	59.4
Undo_Dancer	None	3.8	4.2	18.9	73.1
GT_Fly	None	5.4	9.9	25.2	59.5
Poznan_Street	None	1.6	3.2	10.5	84.7
Poznan_Hall2	None	2.4	3.8	19.6	74.2

Average	None	3.5	5.9	18.8	71.9

**Table 3:** Statistical analysis for accuracies of ET methods in intradepth map coding.

Sequences	ET based on the homogeneity checking	ET based on the texture video-depth map correlation
Kendo	82.3%	91.1%
Balloons	79.5%	88.3%
Newspaper	89.2%	95.8%
Shark	83.5%	92.2%
Undo_Dancer	76.1%	86.8%
GT_Fly	83.8%	90.3%
Poznan_Street	72.2%	84.6%
Poznan_Hall2	87.3%	93.9%

Average	81.7%	90.4%

**Table 4:** Statistical analysis of accuracy for two treeblocks type.

Sequences	Treeblocks in homogeneous region				Treeblocks in complex region			
	QP = 34	QP = 39	QP = 42	QP = 45	QP = 34	QP = 39	QP = 42	QP = 45
Kendo	89%	94%	97%	99%	62%	71%	79%	84%
Balloons	79%	88%	94%	97%	58%	65%	73%	80%
Newspaper	88%	93%	95%	98%	63%	71%	74%	86%
Shark	93%	95%	98%	100%	82%	90%	93%	96%
Undo_Dancer	92%	94%	98%	99%	85%	92%	94%	97%
GT_Fly	95%	97%	98%	100%	79%	87%	92%	94%
Poznan_Street	90%	93%	96%	98%	72%	76%	80%	87%
Poznan_Hall2	92%	96%	97%	98%	69%	75%	82%	89%

Average	90%	94%	97%	99%	71%	78%	83%	89%

**Table 5:** Statistical analysis of accuracy for proposed algorithm.

Sequences	Treeblocks in complex region			
	QP = 34	QP = 39	QP = 42	QP = 45
Kendo	86%	92%	94%	97%
Balloons	78%	85%	90%	94%
Newspaper	83%	87%	93%	96%
Shark	88%	94%	96%	99%
Undo_Dancer	87%	93%	95%	98%
GT_Fly	86%	92%	94%	98%
Poznan_Street	82%	86%	90%	94%
Poznan_Hall2	84%	89%	92%	95%

Average	84%	90%	93%	96%

**Table 6:** Encoder parameters settings.

Codec	HTM4.1
Number of frames	Full length
GOP size	8 (Intrapicture is every 24 frames)
DMM	On
Motion search range	64
MaxCU size	64 × 64
MaxCU depth level	4
Depth QP values	34, 39, 42, 45
Texture QP values	25, 30, 35, 40

**Table 7:** Results of each individual algorithm compared to 3D-HEVC.

Sequences	C2						C3					
	ETMD			AD-CUDRD			ETMD			AD-CUDRD		
	BDBR (%)	BDPSNR (dB)	Dtime (%)	BDBR (%)	BDPSNR (dB)	Dtime (%)	BDBR (%)	BDPSNR (dB)	Dtime (%)	BDBR (%)	BDPSNR (dB)	Dtime (%)
Kendo	1.22	−0.04	−35	0.79	−0.02	−31	1.15	−0.03	−36	0.52	−0.01	−32
Balloons	1.38	−0.05	−31	0.88	−0.02	−28	1.32	−0.04	−32	0.73	−0.01	−29
Newspaper	1.57	−0.07	−37	1.09	−0.04	−25	1.35	−0.06	−39	0.82	−0.03	−25
Shark	0.76	−0.02	−32	0.46	−0.01	−37	0.63	−0.02	−33	0.28	−0.01	−39
Undo_Dancer	0.65	−0.03	−34	0.39	−0.02	−39	0.54	−0.03	−35	0.23	−0.02	−41
GT_Fly	0.89	−0.03	−40	0.62	−0.01	−35	0.72	−0.02	−41	0.41	−0.01	−36
Poznan_Street	1.23	−0.06	−31	1.23	−0.03	−26	1.31	−0.05	−31	0.89	−0.02	−26
Poznan_Hall2	2.12	−0.09	−39	1.38	−0.06	−23	1.92	−0.08	−40	1.02	−0.04	−23

Average	1.22	−0.049	−34.9	0.86	−0.026	−30.5	1.12	−0.041	−35.9	0.61	−0.019	−31.4

**Table 8:** The proposed overall algorithm compared with a state-of-the-art fast intramode decision algorithm in [10].

Sequences	C2						C3					
	Overall algorithm			FDMMS			Overall algorithm			FDMMS		
	BDBR (%)	BDPSNR (dB)	Dtime (%)	BDBR (%)	BDPSNR (dB)	Dtime (%)	BDBR (%)	BDPSNR (dB)	Dtime (%)	BDBR (%)	BDPSNR (dB)	Dtime (%)
Kendo	1.28	−0.04	−45	0.51	−0.01	−29	1.21	−0.04	−47	0.52	−0.01	−30
Balloons	1.53	−0.05	−43	0.54	−0.02	−30	1.47	−0.05	−44	0.49	−0.01	−30
Newspaper	1.64	−0.08	−46	0.89	−0.03	−22	1.62	−0.07	−48	0.79	−0.02	−23
Shark	0.79	−0.04	−44	0.36	−0.01	−25	0.72	−0.03	−45	0.35	−0.01	−26
Undo_Dancer	0.64	−0.04	−45	0.39	−0.02	−24	0.61	−0.04	−46	0.37	−0.02	−24
GT_Fly	0.88	−0.03	−46	0.42	−0.01	−23	0.86	−0.03	−49	0.38	−0.01	−23
Poznan_Street	1.97	−0.07	−41	0.35	−0.02	−28	1.91	−0.06	−42	0.27	−0.02	−29
Poznan_Hall2	2.33	−0.11	−47	0.71	−0.03	−33	2.32	−0.11	−48	0.64	−0.03	−34

Average	1.38	−0.057	−44.6	0.52	−0.019	−26.7	1.34	−0.053	−46.1	0.48	−0.016	−27.4
